# In-Depth Analysis of the Intravitreal Biocompatibility of Polyethylene Glycol of Different Molecular Weight in an In Vivo Porcine Model

**DOI:** 10.1167/iovs.67.2.37

**Published:** 2026-02-19

**Authors:** Maximilian Hammer, Ludwig Geisweid, Niklas Junker, Sabrina Wohlfart, Lea Skrzypczyk, Bryan Calder Ackermann, Jonathan Herth, Alexander Studier-Fischer, Walter Mier, Gerd U. Auffarth, Victor A. Augustin, Philipp Uhl

**Affiliations:** 1 University Clinic Heidelberg, Department of Ophthalmology, Heidelberg, Germany; 2 David J. Apple Laboratory for Vision Research, Heidelberg, Germany; 3 Department of Nuclear Medicine, Heidelberg University Hospital, Heidelberg, Germany; 4 Institute of Pharmacy and Molecular Biotechnology, Heidelberg, Germany; 5 Department of Urology and Urological Surgery, University Medical Center Mannheim, Mannheim, Germany

**Keywords:** polyethylene glycol (PEG), PEGylation, intravitreal injection, sustained drug delivery, intravitreal drug delivery

## Abstract

**Purpose:**

Polyethylene glycol (PEG)ylation is an established, increasingly used method to prolong the half-lives of active compounds applied intravitreally. However, the biocompatibility of high doses of high molecular weight PEG has never been systematically assessed in a translational in vivo model. The aim of this study was to evaluate the intraocular inflammatory and pro-angiogenic response as well as structural and functional retinal alterations after the intravitreal injection of PEG of varying molecular weight injected intravitreally in a large animal in vivo pig model.

**Methods:**

This cohort study includes 12 pigs divided into 4 cohorts. Each cohort received an intravitreal injection of either 0.1 mL with 400 mg/mL of PEG-400, PEG-2000, PEG-40000 or an injection of balanced salt solution (BSS), respectively. Biocompatibility measurements including fundoscopy, optical coherence tomography (OCT) and electroretinography (ERG) were performed prior and 2, 4, and 6 weeks post-injection. Six weeks post-injection, the animals were euthanized, aqueous and vitreous taps were taken for cytokine and signal protein measurements, and immunostainings were performed.

**Results:**

All animals with intravitreal PEG-injections showed significant preretinal vitreous cells, most likely hyalocytes, as evidenced by OCT, but no signs of intraretinal inflammation were seen in the immunostainings conducted 6 weeks after injection. Untreated left control eyes and eyes injected with BSS showed no signs of inflammation throughout the whole study period. Functional and structural retinal parameters showed no significant alterations over the course of 6 weeks. Cytokine analyses showed no prolonged inflammatory reaction.

**Conclusions:**

The one-time intravitreal injection of PEG causes mild intravitreal inflammatory reactions, as evidenced in OCT by increased vitreous cells. However, retinal structure and function is not affected.

Intravitreal injections are an established method to deliver drugs for the treatment of retinal and choroidal diseases.^1^ Most of the currently clinically applied drugs, specifically all approved anti-VEGF agents, are antibodies or fragments of such.^2^ With new developments, such as complement inhibition to treat geographic atrophy,^3^^,^^4^ the relevance of improving intravitreal half-life of small peptide agents, known for their quick clearance from the vitreous cavity, is on the rise.^5^^–^^7^

An established strategy to increase their half-life is the conjugation of the active compound to polyethylene glycol (PEG),^8^^,^^9^ so called PEGylation. PEG is a hydrophilic polyether, composed of repeating ethylene oxide units. The molecular weight and thus its resulting physicochemical and biological properties of PEG is determined by the number of repeating units.^10^ PEGylation of peptides increases the size of the molecule leading to a reduced diffusion-based intraocular clearance.^8^ One prominent representative of PEGylated small peptide molecules is Pegcetacoplan (Syfovre), a US Food and Drug Administration (FDA)-approved complement-factor inhibitor consisting of 2 identical pentadecapeptides conjugated to PEG-40000.^5^

However, PEG is suspected to cause inflammatory reactions and neovascularization.^5^^,^^11^ In vivo studies in small animals confirmed these potential adverse effects showing complement-activation, increased levels of proangiogenic factors, and retinal degeneration.^12^^–^^14^ Furthermore, Abicipar pegol, a small peptide molecule conjugated to a 20 kDa PEG serving as an anti-VEGF-agent, was denied FDA approval after adverse inflammatory reaction in phase 3 trials.^15^ Despite these indications of inflammatory and neovascular reactions to PEG, the biocompatibility of intravitreal PEG in varying molecular weight has never been profoundly studied in a large animal model under fully controlled conditions in wildtype eyes leaving the clinical significance of these observations unclear.

Thus, this study aimed to systematically evaluate the biocompatibility of intravitreal PEG-400, PEG-2000, and PEG-40000 compared with balanced salt solution (BSS)-SHAM-injections in German landrace pigs. The animals were examined using optical coherence tomography (OCT), fundoscopy, full-field electroretinography (ff-ERG), and IOP measurements over 6 weeks. To evaluate retinal inflammation, histopathological analysis including fluorescent immunostaining and immunohistochemistry to detect immune cell infiltration as well as measurement of cytokine and angiogenic factor level in aqueous humor and vitreous humor was performed after euthanasia of the animals.

## Materials and Methods

### Animals

Twelve German landrace pigs, weighing between 30 and 45 kg and aged approximately 4 to 5 months at the start of the study, were included. The pigs were sourced from a local farmer and housed at the Interfaculty Biomedical Research Facility (IBF) of Heidelberg University, with water ad libitum and restricted food access. The facility is overseen by veterinarians. The study received approval by the local ethics committee (ethical approval number: 35-9185.81/G-11/24, Regierungspräsidium Karlsruhe) and adhered with the ARVO guidelines for the use of animals in ophthalmic and vision research. All animals were managed in accordance with German animal welfare laws, EU directives (2010/63/EU), and ARRIVE guidelines.

### Intravitreal Injections

PEG-400, PEG-2000 and PEG-40000 were purchased from Sigma Aldrich Chemie GmbH (Steinheim, Germany). For confirmation of the molecular weight, matrix-assisted laser desorption/ionization (MALDI) analysis was performed. The spectra are presented in Supplementary Figure S1. BSS was purchased from Beaver-Visitec International Ltd. (Bidford on Avon, UK).

**Figure 1.**
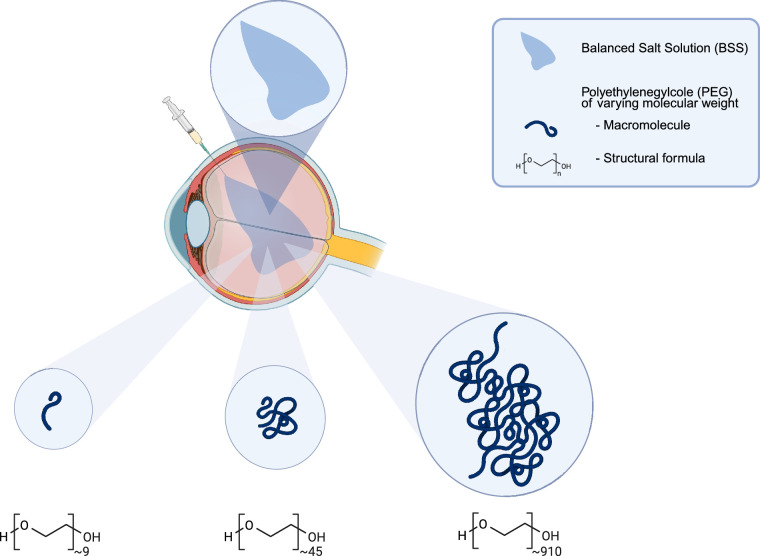
In vivo evaluation of intravitreal inflammatory response and biocompatibility in the porcine model. The 400 mg/mL PEG-400 (*n* = 3), PEG-2000 (*n* = 3), and PEG-40000 (*n* = 3) as well as balanced salt solution (*n* = 3) was administered by intravitreal injection of 0.1 mL at the pars plana area to assess intraocular inflammatory response and biocompatibility over the course of 6 weeks in the eyes of German Landrace pigs.

Intravitreal injections were performed following baseline biocompatibility examinations (see below) in accordance with clinical standards at the pars plana of the right eye. Inject-F 1 mL syringes (B. Braun, Melsungen, Germany) and Zero Residual 33G × 3/8″ needles (SJJ Solutions, Den Haag, The Netherlands) were used to inject 40 mg PEG in 0.1 mL (400 mg/mL) or BSS, respectively, after preoperative conjunctival disinfection with Povidone-iodine solution (Braunol, B. Braun, Melsungen, Germany) for 30 seconds. After injection, the eye was disinfected again with Povidone-iodine solution for 30 seconds and moxifloxacin 5 mg/mL (Vigamox, Novartis, Nürnberg, Germany) and ofloxacin ointment 3 mg/g (Floxal, Bausch+Lomb, Berlin, Germany) were administered topically. The eyes were covered with sterile band aids after the procedure. The experimental setup is shown in Figure 1.

### Biocompatibility Examination

**Figure 2.**
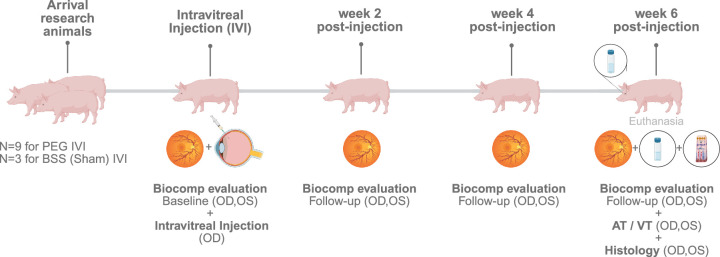
Study protocol. Twelve pigs were divided into four cohorts to assess intraocular inflammatory response and biocompatibility of intravitreal PEG of varying molecular weight (*n* = 3 for PEG-400, *n* = 3 for PEG-2000, and *n* = 3 for PEG-40000) compared to balanced salt solution (*n* = 3) over the course of 6 weeks. After baseline biocompatibility examination in both eyes, intravitreal injection was administered at week 0. Follow-up biocompatibility examinations including OCT, ff-ERG, fundus examination, and IOP-measurement were performed every 2 weeks for 6 weeks in both eyes. After the last biocompatibility examinations 6 weeks post-injection, aqueous and vitreous taps were taken from the pig's anterior chamber for cytokine measurements. Animals were enucleated after euthanization for histological and immunohistochemical examinations. AT, aqueous tap; ff-ERG, full-field electroretinography; IOP, intraocular pressure; OCT, optical coherence tomography; OD, right eye; OS, left eye; PEG, polyethylene glycol; VT, vitreous tap.

Biocompatibility examinations were performed directly prior the intravitreal injection (baseline) as well as 2, 4, and 6 weeks after injection on treated right eyes, as well as on untreated left eyes serving as negative controls. The study protocol is visualized in Figure 2.

For all biocompatibility examinations, the animals were sedated with azaperone (6 mg/kg; Sedanol, WDT, Garbsen, Germany;) and anaesthetized with ketamine (11 mg/kg; CP Pharma, Burgdorf, Germany), and midazolam (2 mg/kg; Hameln Pharma, Hameln, Germany), applied intramuscularly. For maintaining the anesthesia, we used Isoflurane (CP Pharma, Burgdorf, Germany). All biocompatibility examinations were performed in a fully mydriatic, cycloplegic state, using cyclopentolate (0,5%), epinephrine (5%), and tropicamide (1%).

The biocompatibility examination included IOP measurement, OCT, fundus photography, and full-field electroretinography (ff-ERG).

IOP was performed using the iCare Tonovet Plus (iCare Finland Oy, Vantaa, Finland). For OCT scans, we used the Spectralis OCT system (Heidelberg Engineering GmbH, Heidelberg, Germany). OCT scans were placed in the visual streak, the cone-enriched center corresponding to the human macula, and compared using the follow-up function. Fundus photography was taken using the ClearView2 veterinary fundus camera (Optibrand Ltd., Fort Collins, CO, USA).

ERG measurements were performed with the RETevet (LKC Technologies, Gaithersburg, MD, USA) system, following the Dog, Cat, Nonhuman Primate International Society for Clinical Electrophysiology of Vision (ISCEV) 6 Step testing procedure. The grounding electrode was positioned on the pig's forehead, the reference electrode 2 cm temporal to the lateral canthus. The active electrode was placed on the cornea using Carbomer (Vidisic, Dr. Gerhard Mann chem.-pharm. Fabrik GmbH, Berlin, Germany) to enhance adhesion and prevent corneal damage. The performed testing protocol for each eye is shown in Supplementary Table S4. The dark adaption time was 15 minutes.

### Histological Examination and Immunohistochemistry

Detailed methodology and all used compounds are presented in Supplementary Methods S3.

All injected eyes were enucleated postmortem. After filling the vitreous cavity with pre-cooled isopentane application of Tissue Freezing Medium, eyes were frozen in liquid nitrogen. The eyes were cut by cryostat (Leica CM1850, Germany) cooled down to −16°C to −19°C in 5 to 7-µm thick slices and air-dried.

For hematoxylin and eosin (H&E) staining, slides were rinsed in distilled water and then immersed in hematoxylin solution (acidic Mayer’s hematoxylin solution; Carl-Roth GmbH + Co. KG, Karlsruhe, Germany). After bluing and rinsing in distilled water, counterstaining with eosin G solution (0.5%, Carl-Roth GmbH + Co. KG, Karlsruhe, Germany) was performed and the slides were dehydrated in ascending ethanol solutions and covered.

The slides for fluorescent immunohistology were fixed in acetone and air-dried. For blocking, 10% horse serum was used. Anti-CD45 and anti-GFAP primary antibodies in BSA/PBS were applied on the sections, PBS served as negative control. Staining was performed with Cy3-conjugated donkey anti-mouse secondary antibody, DAPI and Phalloidin were applied for nuclear and actin filament staining.

For immunohistochemistry the slides were fixed in acetone, air-dried, and 2.5% horse serum was applied for blocking. Anti-CD45 primary antibody, diluted in BSA/PBS, was applied on the tissue sections. For negative controls PBS was used instead of the primary antibody. Staining was performed using a horse anti-mouse secondary antibody, followed by Diaminobenzidine. Counterstaining was performed with hematoxylin.

H&E and immunohistology stained slides were imaged in the Nikon Imaging Centre Heidelberg, using a Nikon Eclipse Ni-E, equipped with a Nikon DS-Ri2 camera for H&E- and immunohistochemistry staining and a DS-Qi2 camera for immunofluorescent staining.

Preparation of the tissue, imaging and analysis was performed by three different, blinded investigators (authors L.G., L.S., and M.H.).

### Cytokine and Protein Measurements

Aqueous and vitreous taps were taken after euthanization 6 weeks post-injection. The samples were stored at −80°C immediately after collection. We used ELISA to perform measurements of VEGF-A (Porcine VEGF-A ELISA Kit; Sigma Aldrich, Merck KGaA, Darmstadt, Germany), Angiopoietin 2 (Pig Angiopoietin 2 ELISA Kit; Cusabio, Houston, TX, USA), IL-6 (Swine IL-6 ELISA Kit; Thermo Fisher Scientific, Waltham, MA, USA), and TNF-⍺ (Swine TNF-⍺ ELISA Kit; Thermo Fisher Scientific, Waltham, MA, USA). We further measured levels of GM-CSF, IFNγ, IL-1α, IL-1β, IL-1ra, IL-2, IL-4, IL-6, IL-8, IL-10, IL-12, IL-18, and TNFα in aqueous and vitreous taps with MILLIPLEX Porcine Cytokine/Chemokine Magnetic Bead Panel (Sigma Aldrich, Merck, Germany). Kits were used following the manufacturer's instructions.

### Statistical Analysis

For the statistical analysis a paired two-tailed *t*-test was used and compared the right and left eyes in amplitudes of each ERG test, VEGF-level, and cytokine level at the week 6 time point. Values below the limit of detection were imputed as 0. Mean values were calculated for each group as well as the standard error of the mean. GraphPad Prism 10 (GraphPad Software, Boston, MA, USA) was used for all statistical analyses.

## Results

### Intravitreal Injections

All 12 animals underwent uneventful intravitreal injections (see Figs. 1, 2). Specifically, no vitreous hemorrhage, crystalline lens touch, or retinal detachments occurred. No systemic adverse reactions were present; all animals completed the total duration of the experiment of 6 weeks (see Fig. 2).

### Evaluation of Biocompatibility

#### Fundoscopy

**Figure 3.**
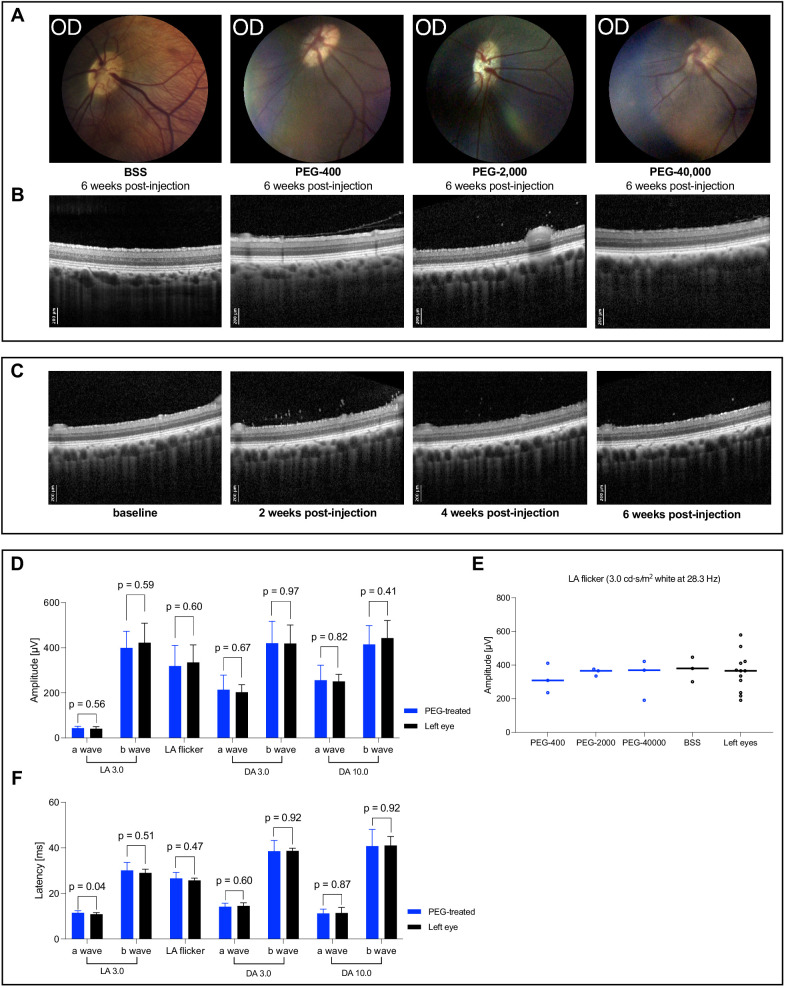
Biocompatibility examinations and evaluation of intravitreal inflammatory response. Over the course of 6 weeks, fundal photography, OCT scans, and ff-ERG were performed every 2 weeks. (**A**) Representative color fundus photographs from one eye of each group 6 weeks post-injection showing no signs of retinal damage and especially no signs of neovascularization or alterations in retinal vasculature. (**B**) Representative OCT scans of the visual streak area of the pigs’ retina from one eye of each group 6 weeks post-injection. In all groups, the presence of vitreous cells was observed. No alterations in retinal microstructure and especially no signs of retinal edema, subretinal fluid or RPE-damage occurred. (**C**) Representative OCT scans of a treated eye from one animal of the PEG-2000 group over the course of 6 weeks. At the 2-week examination, vitreous cells were seen during examination and OCT. The inflammatory reaction, although regredient, was present until the 6 weeks examination. (**D, F**) Amplitudes and latencies of a- and b-waves of all performed ff-ERG-tests 6 weeks post-injection. Comparison of amplitudes and latencies of the treated right eyes from the PEG-groups and paired untreated left eyes showed no significant differences in any of the tests indicating unaffected retinal function in the treated eyes 6 weeks post-injection. (**E**) Amplitudes of LA flicker test (3.0 cd⋅s/mm^2^ at 28.3 Hz) of the right eyes from the different PEG-treated groups compared to BSS-treated right eyes and all untreated left eyes showed no significant difference in any of the PEG-groups indicating uncompromised retinal function compared to BSS-treated and untreated eyes. Fundus photographs were brightened after acquisition due to the dark fundus of the animals. DA, dark adapted; ff-ERG, full-field electroretinography; LA, light adapted; OCT, optical coherence tomography; PEG, polyethylene glycol; RPE, retinal pigment epithelium.

In all 12 treated pigs, no morphological changes were apparent during fundoscopy compared with baseline and untreated left eyes, respectively, over the course of 6 weeks. The eyes showed no cataract development with few vitreous cells quantified using OCT in all eyes injected with PEG irrespective of the molecular weight, an optic disc with sharp margins, and vital coloration. The retina was attached in all quadrants. The retinal vasculature appeared normal with no exudates or signs of neovascularization (Fig. 3A).

### Optical Coherence Tomography

The OCT scans of all animals in the three PEG-cohorts showed vitreous body cells of varying degrees near the posterior pole in the treated right eyes. We suspect these cells most likely to be hyalocytes, although cytological proof was not performed. The vitreous body cells appeared first in the 2-week examination and decreased after the following 4 weeks with still remaining vitreous cells in some animals at the 6-week examination (Fig. 3B). The most severe vitreous reaction occurred in the animals treated with PEG-2000 intravitreally (Fig. 3C).

Moreover, in 7 of 9 treated right eyes in the PEG-cohorts, separation of the posterior vitreous cortex from internal limiting membrane (posterior vitreous detachment [PVD]) occurred in either week 4 or week 6 post-injection. Only one eye treated with BSS showed PVD, untreated fellow eyes did not show PVD.

None of the BSS-treated eyes or untreated left eyes showed signs of vitreous reaction (see Fig. 3B). Retinal thickness was not altered in PEG or BSS treated eyes. The retinal thickness is displayed in Supplementary Figure S2.

### Electroretinography

The ERG showed no significant difference in a- and b-wave amplitudes between the treated right eye and the untreated left eye in all performed test at the 6 weeks examination (Fig. 3D). The amplitudes of the light adapted 3.0 cd⋅s/m^2^ 28.3 hertz (Hz) flicker test measured in the treated right eye of all PEG-cohorts as well as in the BSS-treated right eyes were equivalent to the amplitudes of all untreated left eyes (Fig. 3E). Over the course of 6 weeks, no significant differences in amplitudes or latencies in all tests appeared among PEG-treated right eyes, BSS-treated right eyes, and untreated left eyes.

### Histology and Immunohistochemistry

**Figure 4.**
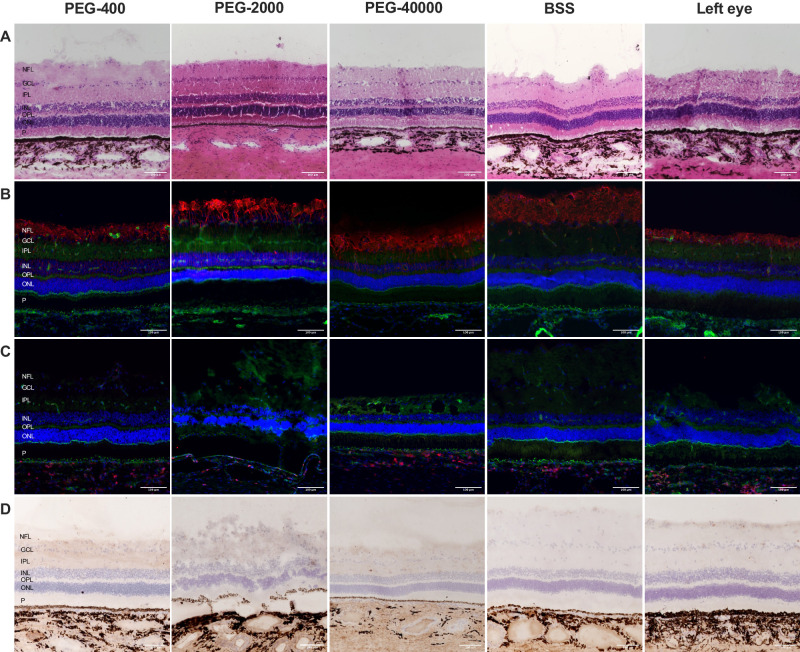
Histological and immunohistochemical analysis of the retina of eyes with injection of PEG-400, PEG-2000, PEG-40000, BSS, and untreated left eyes 6 weeks post-injection. Magnification: × 100 (**A**) Hematoxylin and eosin (H&E) staining of representative retinal sections. Retinal layers are annotated exemplarily for the animal with PEG-400 injection. No alterations in retinal cellular structure are visible. (**B,** **C**) Immunofluorescent staining of glial cell and leucocyte markers. DAPI staining (*blue*) was performed to visualize nuclei, Phalloidin staining (*green*) indicates actin cytoskeleton in all panels. (**B**) Staining of GFAP (*red*) showed typical distribution in the inner retina with no signs of notable Müller-cell activation in all treated and untreated eyes. (**C**) Staining of CD45 (*red*) showed tissue resident leucocytes but no intraretinal leucocyte infiltration in any of the examined eyes. (**D**) Immunohistochemical staining for CD45 also showed no intraretinal leucocyte infiltration in all treated and untreated eyes. ELM, external limiting membrane; GCL, ganglion cell layer; GFAP, glial fibrillary acidic protein; INL, inner nuclear layer; IPL, inner plexiform layer; NFL, nerve fiber layer; ONL, outer nuclear layer; OPL, outer plexiform layer; P, photoreceptor layers.

H&E histology revealed normal retinal morphology after 6 weeks in all studied eyes, specifically no loss of cell density, signs of cytotoxicity, or atrophy occurred in H&E staining (Fig. 4A).

Staining of GFAP showed normal distribution in the inner retinal layers in all analyzed sections with no remarkable differences between the PEG-cohorts and the controls indicating no significant glial proliferation and activation (Fig. 4B).

Immunohistochemical and immunofluorescent staining of CD45 showed no pathological intraretinal signal in any of the PEG-treated eyes as well as in the BSS-treated and untreated controls indicating no leucocyte infiltration at the 6 weeks post-injection time point (Figs. 4C, 4D).

### Cytokine and Signal Protein Measurements

**Figure 5.**
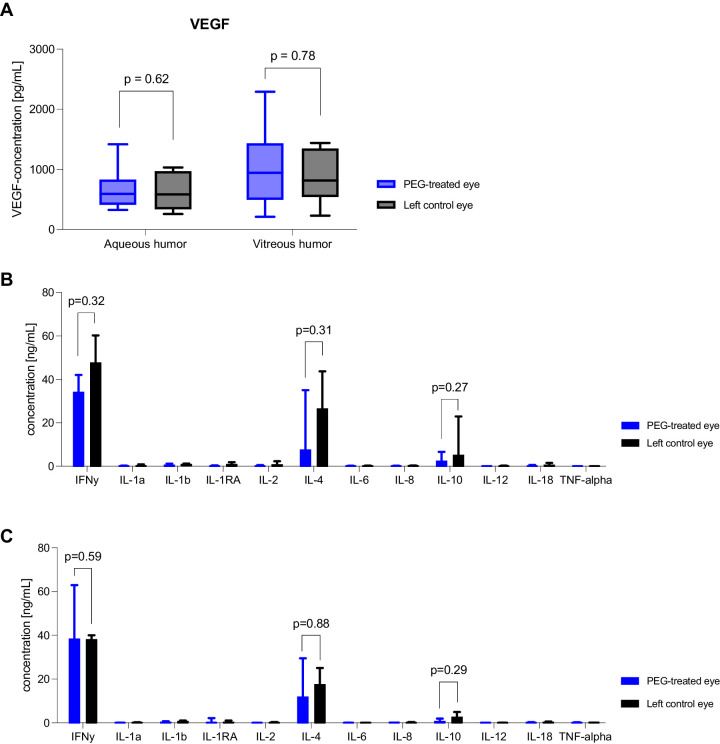
VEGF and cytokine measurements 6 weeks post-injection from aqueous and vitreous taps. (**A**) Levels of VEGF showed no significant differences between PEG-treated right eyes and untreated left eyes in both aqueous and vitreous humor. Cytokine levels in both aqueous (**B**) and vitreous (**C**) humor were low, in some cases below the limit of detection. IFNγ, IL-4, and IL-10 showed the highest measurable levels. No significant differences between PEG-treated right eyes and untreated fellow eyes in aqueous or vitreous humor occurred in any of the measured cytokines.

ELISA-based quantification of VEGF in aqueous and vitreous taps of PEG-treated eyes showed no significant elevation in levels compared with untreated fellow eyes 6 weeks post-injection (Fig. 5A). Levels of angiopoietin-2 were above detection levels in only two of the analyzed samples, therefore no significant statistical impact was observed.

The levels of all cytokines measured with multiplex bead assay in both aqueous taps and vitreous taps 6 weeks post-injection remained low across all groups, in some cases below the limit of detection. There were no significant differences in cytokine levels between PEG-treated eyes and untreated fellow eyes (Figs. 5B, 5C).

These findings indicate no sustained upregulation of pro-angiogenic, or pro-inflammatory pathways, and no intravitreal secretion of cytokines and chemokines related to either PEG or the injection itself 6 weeks post-injection.

## Discussion

In this study, we evaluated the in vivo biocompatibility as well as the inflammatory and pro-angiogenic response in eyes after intravitreal injection of PEG of different molecular weight in a large animal model over 6 weeks. We showed all molecular weights of PEG to cause a distinctive, variable vitreal reaction for at least 6 weeks post-injection. Although intravitreal inflammatory response occurred following PEG-injection, no intraretinal inflammation could be objectified. Structural integrity and function of the retina remained unaffected. Immune reaction could not be quantified by cytokine measurements in aqueous and vitreous taps 6 weeks post-injection, as well as no pro-angiogenic tendencies appeared neither morphologically nor biochemically.

PEG is long suspected to have impact on ocular structures and to cause inflammatory reactions. As rabbits are an established model in eye research, previous studies investigated ocular biocompatibility of PEG in rabbits with varying results. Although ethylene glycol monomers showed adverse effects on the rabbits eye even when administered orally,^16^ PEG-400 was revealed to cause structural damage on ocular tissues like cataract or atrophy of retinal pigment epithelium (RPE) with functional damage in ff-ERG when administered in the anterior chamber.^14^ After intravitreal application, recent studies reported biocompatible results in rabbits with no signs of intraocular inflammation.^17^ In rodents, administration of PEG was shown to induce retinal degeneration^18^ and formation of neovascularization,^12^ observations which were questioned recently.^13^

In both rodent and rabbit models, the impact of PEG on ocular tissue, if observed, was described to be structurally and functionally relevant manifesting in atrophy, retinal thinning, and neovascularization even after a single application. In contrast, adverse results in humans were not highly prevalent, which is in accordance with the data presented in this study. This strengthens the role of large animal pig models as a more robust animal model in translational ophthalmic research.

Although the limited inflammatory response leaving retinal structure and function unaffected, long-term effects of PEG exposure may induce greater severity. Studies regarding the reaction to PEG when applied systemically described increased clearance of PEGylated drugs after repeated administration.^19^ This effect called accelerated blood clearance (ABC)^20^ was recently attributed to PEG-specific antibodies,^21^^,^^22^ produced after the first dosage of the PEGylated drug^23^^,^^24^ or existing preformed.^25^ These PEG-specific antibodies were also made evident to cause hypersensitivity-reactions via complement activation (CARPA).^26^^,^^27^ This effect was described specifically in porcine models causing pseudo-anaphylaxis after repeated systemic intravenous injection of PEGylated liposomes.^28^

Considering these data on repeated administration of PEGylated drugs, the intravitreal reaction observed in this study may be interpreted as a primary immune reaction to PEG. Although the intravenous half-life of pegcetacoplan is only approximately 12 days,^29^ the intravitreal half-life is expected to be much higher, due to the reservoir function of the vitreous.^7^ Subsequent injections could therefore cause hypersensitivity reactions even after a single application. Numerous injections in a high application frequency over the course of years could therefore cause chronic and increasing inflammatory reactions, which may result in retinal degenerative diseases or retinal neovascularization.^30^^,^^31^ This aligns with the higher conversation rate from dry to exudative macular degeneration in pegcetacoplan treated patients with monthly injections compared with injections every other month.^5^

Whereas PEG of all molecular weights caused intravitreal reaction, degree and duration varied among the three groups. Eyes treated with PEG-2000 presented the highest number and longest duration of vitreal cells. This indicates an association between molecular weight and immunogenicity. Previous studies regarding the systemic application of PEG suggested multiple small PEG-molecules approximately 2 kDa to induce higher titers of anti-PEG antibodies than single PEG molecules approximately 40 kDa.^23^^,^^32^^–^^34^ Furthermore, most of the cases of immediate-type hypersensitivity reaction to PEG after systemic administration are reported for molecular weights between 3 and 6 kDa.^35^ For intravitreal application, Abicipar pegol, PEGylated with 20 kDa PEG has been associated with a substantially higher incidence of treatment emergent anti-drug antibodies and intraocular inflammation compared to pegaptanib, PEGylated with 40 kDa PEG.^36^ This indicates an inverse correlation between molecular weight and immunogenicity. Aside from that, the lower vitreal reaction to PEG-400 compared with PEG-2000 could be explained by faster intraocular clearance for molecules with lower molecular weight.^37^

The clinically approved doses of intravitreally administered PEGylated drugs is 15 mg for pegcetacoplan,^5^ 2 mg for Abicipar pegol,^38^ and 0.3 mg for pegaptanib.^39^ In contrast, the dose of 40 mg pure PEG used in this study was considerably higher. As the injected volume of 0.1 mL is relatively low and PEG 400 appears as a clear fluid, we decided to administer this elevated dose to avoid missing biocompatibility issues due to an insufficient amount applied. This must be taken into account when interpreting the inflammatory reaction observed in this study, knowing the titer and half-life of anti-PEG-antibodies formed after systemic administration being dose dependent.^40^

In this study, we only tested three molecular weights of PEG for intravitreal application. We chose to evaluate PEG-400 as the shortest PEG molecule due to the results of previous investigations in rabbits^14^ and PEG-40000 as upper limit referring to pegcetacoplan. As we thought PEG-20000 to deliver similar results as PEG-40000, and in order to fulfill the 3R principle in animal testing, we decided to investigate the intermediate-length PEG-2000 as the most commonly used molecular weight in systemic applications instead. Regarding the clinical data on this matter, future studies should aim to elaborate the difference in immunogenicity of those intravitreal applied PEG molecules.

Of course, this study holds inherent limitations. Here, PEG was only injected once at the beginning of the study period. The inflammatory reaction may increase after repeated injections, which could not be depicted by our study. Further, aqueous and vitreous taps, as well as enucleation followed by histological examination, were only performed 6 weeks post-injection. Possible humoral or cellular immune responses intraretinal, intravitreal, or in the anterior chamber could not be quantified at different time points. However, we decided to not perform aqueous taps and other interventions during the study period to reduce the risk of the interventions confounding results.

In summary, this study showed that intravitreally injected PEG of all molecular weights causes a mild intravitreal inflammatory reaction not affecting retinal structural and functional integrity when applied once. This in vivo porcine data gathered under controlled conditions in healthy eyes aligns with data on higher inflammatory rates for PEGylated drugs in human geographic atrophy trials. Further research must be conducted to unravel the effect of repeated injections of PEG and to quantify the influence of PEG molecular weight on inflammatory reactions.

## Supplementary Material

Supplement 1
