# Supplementary material for: In-Depth Analysis of the Intravitreal Biocompatibility of Polyethylene Glycol of Different Molecular Weight in an In Vivo Porcine Model
Source: Invest Ophthalmol Vis Sci. 2026 Feb 19;67(2):37. doi: 10.1167/iovs.67.2.37 (PMC12924145; doi:10.1167/iovs.67.2.37)
Supplement: Supplement 1 [file iovs-67-2-37_s001.pdf]

Supplementary Material

Figure S1: Chromatograms of MALDI measurement.

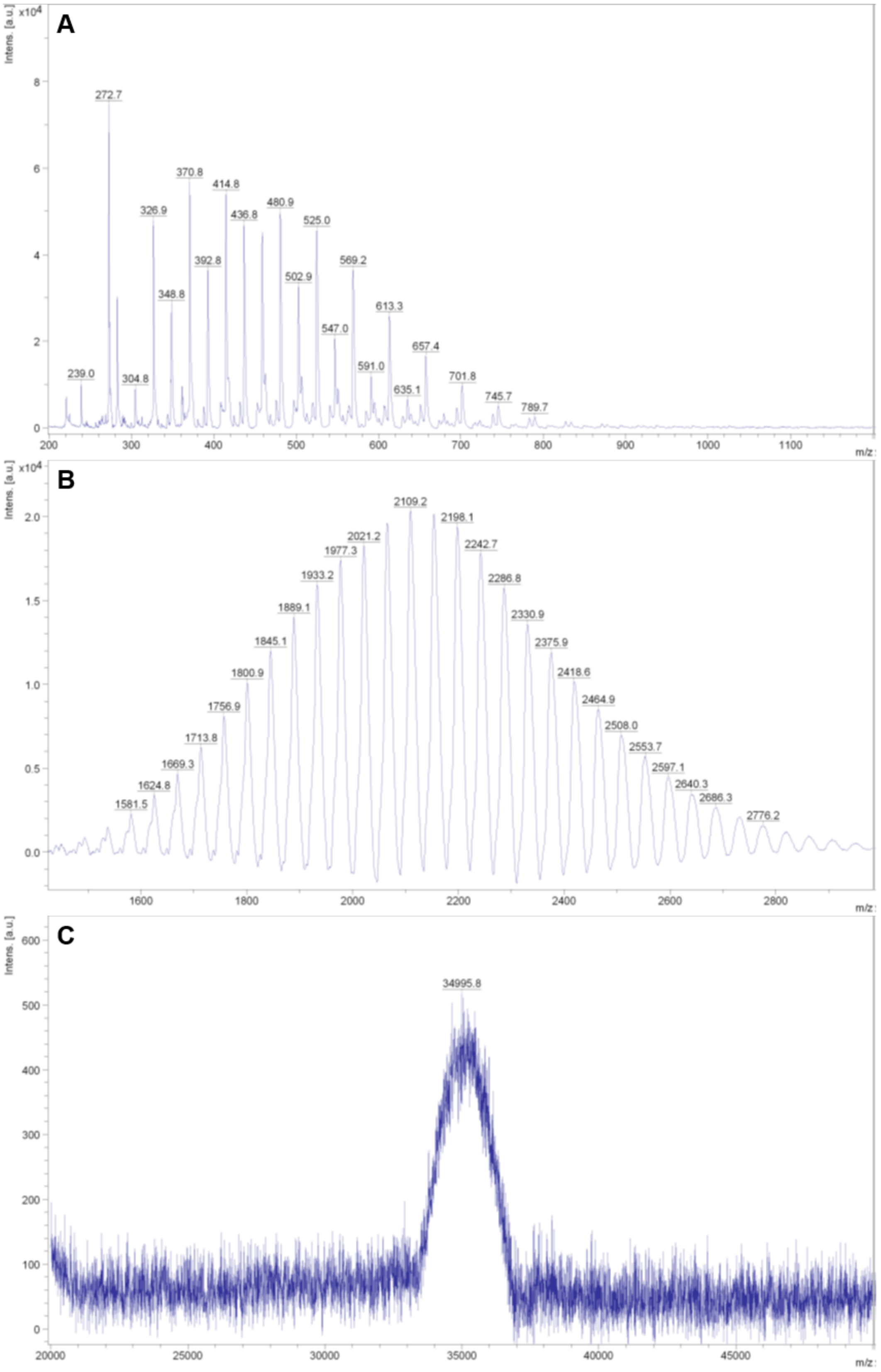

As Matrix in all samples 2,5-Dihydroxybenzoeic acid was used. **A:** PEG400 with a measured molecular weight $M_{W,m}$ = 414.8 g/mol, **B:** PEG2000 with $M_{W,m}$ = 2109.2 g/mol and **C** PEG40000 with $M_{W,m}$ = 34995.8 g/mol.

Figure S2: Total retinal thickness of PEG-treated and untreated fellow eyes.

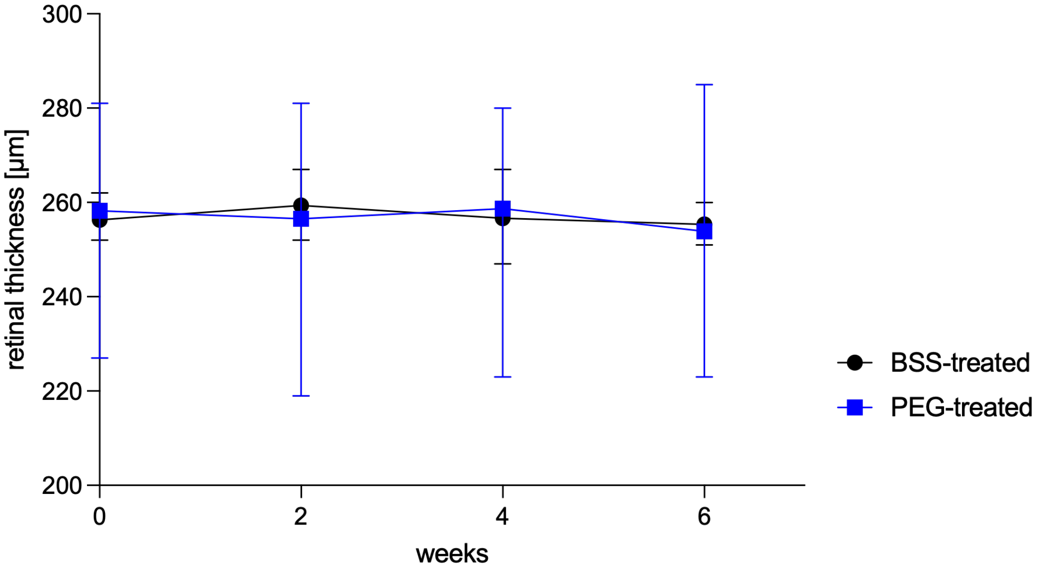

Total retinal thickness showed no significant alterations in both PEG-treated (blue) and BSS-treated (black) right eyes over the course of 6 weeks.

Methods S3: Detailed methodology and used materials for histology and immunohistochemistry

For the Histological examination and immunohistochemistry all injected eyes were enucleated post-mortem. Liquid nitrogen pre-cooled isopentane (>99%, Carl-Roth GmbH + Co. KG, Karlsruhe, Germany) was used to fill the posterior eyecup and Tissue Freezing Medium (Tissue Freezing Medium, LEICA Biosystems, Nußloch, Germany) was applied into the eyecup before freezing the sample in liquid nitrogen.

The eyes were cut by cryostat (Leica CM1850, Germany) cooled down to -16 to -19°C in 5-7 µm thick slices, which were mounted on Superfrost Plus microscopic slides (Epredia, Germany) and air-dried.

The slides for HE staining were rinsed in distilled water and then immersed in hematoxylin solution (acidic Mayer´s hematoxylin solution, Carl-Roth GmbH + Co. KG, Karlsruhe, Germany) for six minutes. Afterwards they were blued in running tap water for 15 minutes, then rinsed in distilled water for two minutes. For three minute Counterstaining we used eosin G solution (0.5%, Carl-Roth GmbH + Co. KG, Karlsruhe, Germany) which was supplemented with one drop of glacial acetic acid (99.8 – 100%, Bernd Kraft GmbH, Den Haag, Netherlands). The slides then were rinsed with tap water one final time, dehydrated in ascending ethanol solutions (70%, 96% and 100%; ZENTRALBEREICH Neuenheimer Feld, Heidelberg, Germany) and cleared in ROTIHistol (Carl-Roth GmbH + Co. KG, Karlsruhe, Germany). For coverslipping we used ROTIHistokitt (Carl-Roth GmbH + Co. KG, Karlsruhe, Germany) and glass coverslips (24 x 50 mm, borosilicate glass, VWR International, Radnor, USA).

The slides for Immunohistology were fixed in Acetone (>99,5%, Carl-Roth GmbH + Co. KG, Karlsruhe, Germany) at -20°C. Afterwards the sections were air-dried, and each section was circled using a hydrophobic barrier pen (PAP pen, Sigma-Aldrich/Merck, Taufkirchen, Germany). For blocking we applied 10% horse serum (S-2000, Vector Laboratories, Newark, CA, USA), diluted in 1% BSA/PBS and let it rest for 30 mins in a dark humid chamber. Subsequently the blocking was removed by tapping the slides onto a tissue. Primary antibodies were diluted in 1% PBS (1:100): anti-CD45 (clone K252.1E4, Bio-Rad Laboratories, Hercules, CA, USA) and anti-GFAP (clone GF 12.24, PROGEN, Heidelberg, Germany) and one control vial with PBS were applied, each on one slice. The slides were stored at 4°C overnight.

On the following day the slides were washed twice in 1% PBS for five minutes. Afterwards the fluorochromes were applied: Cy3-conjugated donkey anti-mouse secondary antibody (Jackson ImmunoResearch Laboratories, West Grove, USA), DAPI (D9542, Sigma-Aldrich/Merck, Taufkirchen, Germany) and Phalloidin (BioLegend, San Diego, CA, USA) were all together diluted in concentrations of 1:200, 1:1000 and 1:100 respectively. The slides then were again stored in a dark humid chamber for 30 mins at room temperature. Following that, we washed the slides again twice for 5 mins, with lightproof covering. At last, all slides were covered with glass coverslips (24 x 50 mm, borosilicate glass, VWR International, Radnor, USA) and VectaMount (Vector Laboratories, Inc., Newark, CA, USA).

For Immunohistochemical staining, slides were fixed in pre-cooled acetone (>99,5%, Carl-Roth GmbH + Co. KG, Karlsruhe Germany) at -20°C for 10 minutes. Afterwards, they were air-dried and subsequently immersed in 3% H_2O_2 (30%, Carl-Roth GmbH + Co. KG, Karlsruhe Germany) in 70% Ethanol (≥70%, Carl-Roth GmbH + Co. KG, Karlsruhe Germany) for 10 minutes covered from light to deactivate endogenous peroxidase. Slides were then washed in distilled water, and the tissue sections were circled with a hydrophobic barrier pen (PAP pen, Sigma-Aldrich/Merck, Taufkirchen, Germany). Blocking was performed with 2.5% horse serum (ImmPRESS® HRP Horse Anti-Mouse IgG Polymer Kit, Vector Laboratories, Inc., Newark, CA, USA) for 30 minutes in a dark humid chamber at room temperature. Horse serum was removed by gently tapping the slides on tissue paper. Anti-CD45 primary antibody (clone K252.1E4, Bio-Rad Laboratories, Hercules, CA, USA) was applied diluted 1:100 in PBS and incubated over night at 4°C in a dark humid chamber.

On the next day, slides were washed twice in PBS for five minutes each. Horse anti-mouse (ImmPRESS® HRP Horse Anti-Mouse IgG Polymer Kit, Vector Laboratories, Inc., Newark, CA, USA) secondary antibody was applied and the slides were incubated for 30 minutes at room temperature in a dark humid chamber. The slides were then washed twice for five minutes in PBS. Diaminobenzidine (DAB Substrate Kit, Peroxidase, Vector Laboratories, Inc., Newark, CA, USA) was applied for four minutes, followed by washing in distilled water. For counterstaining the slides were immersed in hematoxylin for 40 seconds and subsequently blued under running tap water. All slides then were covered with coverslips (24 x 50 mm, borosilicate glass, VWR international, Radnor, USA) using VectaMount mounting medium (Vector Laboratories, Inc., Newark, CA, USA).

When fully dried, HE and Immunohistology stained slides were imaged in the Nikon Imaging Centre Heidelberg, using a Nikon Eclipse Ni-E, equipped with a Nikon DS-Ri2 camera for HE- and Immunohistochemistry staining and a DS-Qi2 camera for Immunofluorescent staining.

Table S4: Overview over the performed ff-ERG tests.

Test description	Adaption	Flash luminance energy	Backgr. luminance	Nr of flashes
LA 3.0	Light	3 cd·s/m^2 at 2 Hz	30 cd·s/m^2	20
LA flicker	Light	3 cd·s/m^2 at 28.3 Hz	30 cd·s/m^2	141 - 424
DA 0.01	Dark	0.01 cd·s/m^2 at 0.5 Hz	off	3
DA 3.0	Dark	3 cd·s/m^2 at 0.1 Hz	off	3
DA 10.0	Dark	10 cd·s/m^2 at 0.05 Hz	off	3
